# Dentistry, Practical and Theoretical

**Published:** 1896-09

**Authors:** William Shaw Twilley

**Affiliations:** Baltimore, Md.


### DENTISTRY, PRACTICAL AND THEORETICAL.[1]

BY WILLIAM SHAW TWILLEY, BALTIMORE, MD.

MR. PRESIDENT AND GENTLEMEN,—I wish to call your attention this evening to dentistry, practical and theoretical, the happy combination united, the condition separated, and the result when antagonized. A man who is a cultured, intelligent, "up-to-date" dentist, at home on every topic scientific, philosophic, well versed in theory, yet using his instruments with ease and dexterity; a skilful, mechanical operator; a man so endowed is considered by some a *rara avis*, yet such men exist,—a striking example to "remind us" "knowledge is power." Over these brilliant men theory exercises a potent fascination, and to them we owe the elevation of the profession to the high plane upon which it stands to-day; but they are comparatively few in number. Such is one I have in mind at present, a Chesterfield in manner and a dentist of rare excellence. He told me he would rather write an essay, the theory, as it were, of filling a tooth, twice over than perform the operation; but, said he, "I realize the importance of practical work, and I am still making every effort to perfect myself in that branch of the profession." That man is my ideal dentist. If perfect theoretically, strive to become so practically, and *vice versa*. But when practice and theory are separated, the dentist, college bred, the proud possessor of the honors of his class, has the theory all in his head, but not in his fingers,—"those four crafty fingers and a thumb,"—what wonderful power lies in those delicate members!

Why does the man whose theoretical knowledge is unassailable fail? He has no practical skill; or, if he possesses the talent, pays no special attention to its development. I can recall a member of the profession, an author of recognized ability, yet, as a good practical man, said to be a dismal failure. Possessing but little me-

---

Black. The fact that a daily antiseptic wash prevents this irritation demonstrates very clearly the cause, and that it has nothing to do with free mercury supposed to be in the coloring substance (vermilion) of the rubber.—[ED.]

[1] Read before the Central Dental Association of Northern New Jersey, May 18, 1896.

chanical talent, he does not realize his mistakes, and his prominence is based on his theoretical knowledge; yet he has succeeded financially. Such is the value of an author's fame. In marked contrast was a young man, whose operations and plate work were a pleasure and a dream, the most delicate, complicated gold work accomplished with rare skill, and yet failed year after year to pass the theoretical barrier raised by the Faculty of the College as a gauge of perfection necessary to enter the profession of dentistry. Ofttimes theory is antagonized by some old experienced dentist, conservative in his ideas, a man who has not kept step with the march of professional advancement. A young graduate arrives upon the scene fresh from his Alma Mater, and electrifies the people with his new wonderful theories of crown- and bridge-work and contour fillings; stigmatizes the dentist who has worked for his father and perhaps his grand-sire as an "old fogy," and causes the old practitioner who has grown gray in the honest practice of his profession to look upon him with the eye of suspicion and ill-concealed aversion. I find, upon inquiry, a majority of the young men who enter colleges east make this frank avowal: "I thought I could make more money out of dentistry than on the farm or in the shop;" or, "There is no dentist in our neighborhood, and I thought I could make a living." This is "poor timber," a bad foundation. What structure can we hope to build from such material? Struggling through their examinations, they start out to make money, no thought of theory or perfect practical work enters into their calculations. Such men often degenerate into mere charlatans, advertising impossibilities, and offering what they term the finest work in dentistry on the instalment plan. This class of dentists is composed largely of three grades of men,—the theoretical without mechanical ability, the mechanical with no theoretical capacity, and the young man who merely managed to pass his examinations.

The colleges are making every effort to raise the standard of dentistry to a profession second to none; to make the degree D.D.S. an open sesame to every portal, and with all my heart I wish them God speed. This noble ambition exists in all reputable colleges, and the State boards supplement and incite them to continue their efforts. It was quite popular in days gone by to ridicule the idea of theory and culture, or, to use a familiar term, "book-learning," in a dentist. Dentistry was considered merely a mechanical art; anybody could be a "tooth-carpenter;" but that day is no more; the people of the present demand that the dentist shall be an "up-to-date" man.

The high plane upon which the profession stands to-day, the recognition it has secured, is due to the energy, the subtle pen, the deep researches of our theoretical brothers. Their work is not completed when the sun goes down, but in the still hours they burn the "midnight oil," searching for the elusive little microbes, bacteria, and other scientific whys and wherefores that trouble the oral cavity. To these eminent dentists we owe a debt of gratitude. What "mountains of paper" and "rivers of ink" have been used by this earnest body of men to elevate and perfect this branch of the healing art! These theoretical dentists are like generals commanding armies; they plan, manœuvre, theorize, but upon whom does the battle fall? On the "rank and file,"—those men who execute the commands of the generals. It is the quiet, hard-working little fellow, who is afraid of the sound of his own voice in the association meetings, but toiling day by day with cunning dexterity executing the difficult handiwork which the great theoretical teacher pictures in "thoughts that breathe and words that burn."

Ah! dentistry is easy work theoretically. If I were to tell you how to construct the most beautiful piece of bridge-work in theory, the lines drawn closer than a hair's breadth, the strength unassailable, articulation rivalling nature's best, and as to cleanliness, it cleans itself, how many before me to-night could make such a piece, a triumph in works of the dental art? I answer but few, indeed, as I am satisfied in my own mind that no progressive workman, and we all aim to be progressive, is ever completely satisfied with the operation, even when finished to the best of his ability.

As I am deeply interested in all that appertains to the advancement of our profession, may I be permitted to digress from my subject in a few remarks advocating the following suggestion: Dentistry would be much benefited if the profession would give more attention to the generous proposal of the "Army Medical Museum and Library," of Washington, D. C. This institution, which enjoys a world-wide reputation, has offered a room free for the exhibition of specimens of dental art. What a wide field of usefulness lies fallow! Why do not the dentists take advantage of this golden opportunity, and by sending some good specimens of our professional skill to this museum successfully demonstrate to the thousands of yearly visitors the value of dental hygiene as a branch of the healing art?

In elevating the standard of dentistry so high, and placing the title on a level with other learned professions, there is another thought that should be taken into consideration: should not every candidate for the degree of D.D.S. possess not only a cultured mind, but a skilful hand in manipulation. Of what advantage is all this theory, if we fail in the performance? Why find the germs of caries, if we cannot destroy? Why teach pathology, if we have no therapeutic results? Therefore, though elevating the standard let us be consistent, not raise it on one side too suddenly, or the good old ship "Dentistry" may capsize with its unevenly balanced cargo.

It was one-sided for many long years, and encountered some adverse winds, but it now seems on fair sea, so let us trim the ship and sail with grace into the beautiful harbor of "Consistency," with "Theory" as our captain, "Practice" as first officer, and as an anchor "Peace and good will to all."

